# Association of plasma arachidonic acid levels with a bipolar disorder and the effects of a FADS gene variant

**DOI:** 10.1038/s41398-024-03141-1

**Published:** 2024-10-14

**Authors:** Takuma Ashizawa, Takeo Saito, Tomo Okochi, Kohei Ninomiya, Kenta Ito, Rei Aoki, Masashi Ikeda, Nakao Iwata

**Affiliations:** 1 Department of Psychiatry, Fujita Health University School of Medicine, Toyoake, Aichi 470-1192 Japan; 2 Department of Psychiatry, Nagoya University Graduate School of Medicine, Nagoya, Aichi 466-8550 Japan

**Keywords:** Bipolar disorder, Pathogenesis

## Abstract

Recent genome-wide association studies (GWASs) have identified fatty acid desaturase (*FADS*) genes, which code key enzymes involved in polyunsaturated fatty acid (PUFA) desaturation as susceptibility genes for bipolar disorder (BD). Several quantitative changes in PUFAs suggest their involvement in BD pathogenesis. Therefore, this study aimed to clarify the relationship between BD and PUFAs by conducting lipidomics covariating with the *FADS* gene variant (rs174550), which is associated with PUFA levels and BD susceptibility. The concentrations of 23 fatty acids were measured using plasma samples from the BD group (*n* = 535) and the control group (*n* = 107). Differences in each PUFA concentration ratio were compared between the two groups. Also, differences in each PUFA concentration ratio were compared for each genotype in rs174550. Our results showed that the BD group had significantly lower concentrations of linoleic acid (LA) (β = −0.36, *p* = 0.023) and arachidonic acid (AA) (β = −0.18, *p* = 0.013) than the control group. Concerning the effect of *FADS* on the PUFA concentration ratio, carriers of C-allele at rs174550 had significantly decreased γ-linolenic acid and AA concentration ratios. A previous GWAS reported that the presence of a C-allele at rs174550 increased the BD risk. This direction is consistent with the lipidomic results of the present study. In conclusion, both the *FADS* and BD were considered to regulate the AA concentration. Thus, as the *FADS* gene variant is crucial for conducting lipidomics of BD we believe that the allele frequency of *FADS* must be analyzed.

## Introduction

Bipolar disorder (BD) is characterized by mood swings, with alternating mania and depression. Among mental disorders, the heritability of BD is approximately 70–80%, as high as that of schizophrenia [1, 2]. Mood stabilizers such as lithium salts and second-generation antipsychotics are used to treat BD; however, their pharmacological mechanism has not yet been elucidated [3, 4]. Moreover, the pathophysiology of BD is still unclear; thus, its elucidation is essential for the development of new treatments and prevention of BD.

Polyunsaturated fatty acids (PUFAs, n-3 PUFAs including α-linolenic acid [ALA], eicosapentaenoic acid [EPA], and docosahexaenoic acid [DHA] and n-6 PUFAs including linoleic acid [LA], γ-linolenic acid [GLA], dihomo-γ-linolenic acid [DGLA], and arachidonic acid [AA]) are possible novel treatment options. This speculation is based on several epidemiological, clinical, biomarker, and genomic evidence. For example, Noaghiul et al. reported a negative correlation between fish consumption and the lifetime prevalence of BD [5]. In addition, clinical trials of PUFA supplementation in patients with BD have been conducted because n-3 PUFAs are safe and convenient to use. However, a clear conclusion has not been established. A recent meta-analysis showed that n-3 PUFA supplementation has beneficial effects on improving BD symptoms [6].

Lipidomics studies have reported that individuals with BD have a PUFA profile different from healthy controls [7–14] although reports on the association between BD and changes in PUFA levels are not always consistent: Chiu et al. reported low plasma concentrations of AA and DHA in patients with BD, whereas Evans et al. revealed no significant difference in the plasma concentrations of AA, EPA, and DHA between the BD group and the control group. Koga et al. analyzed the largest sample size, reporting high plasma concentrations of n-6 PUFAs such as AA and low levels of n-3 PUFAs such as EPA and DHA in patients with BD. Such conflicting results are possibly caused by the small samples analyzed in each study, introducing inadequate statistical power.

Each of the n-3 and n-6 PUFA was biosynthesized by fatty acid desaturase (FADS1 and FADS2). ALA was metabolized to EPA and DHA by the FADS enzyme. Similarly, LA was metabolized to GLA, DGLA, and AA by the FADS enzyme (Supplementary Fig. 1). Recent genome-wide association studies (GWASs) have identified fatty acid desaturase (*FADS1* and *FADS2*) genes, which encode enzymes involved in n-3 and n-6 lipid biosynthesis pathways, as susceptibility genes of BD (top single nucleotide polymorphism [SNP] rs28456) [15, 16]. In addition, many studies of quantitative trait loci (QTL) have shown that *FADS* genes are associated with PUFA concentrations [17–19]. In particular, Drajoo et al. reported that the concentrations of n-6 PUFAs (LA, GLA, DGLA, and AA) and n-3 PUFAs (ALA and EPA) were significantly related to *FADS* gene variants (i.e., LA rs174547, GLA rs174546, DGLA, rs174548, AA rs174577, ALA rs509360, and EPA rs174538). The effect size of variants on increasing or decreasing PUFA levels is modest compared with that of susceptibility for complex disorders, including BD. Interestingly, specific alleles that are associated with each PUFA levels are in tight linkage disequilibrium with BD susceptibility variants (i.e., the G allele of rs28456), indicating that low AA levels may be associated with susceptibility to BD.

Taken together, we speculate that the inconsistent results of lipidomics for BD may be induced by not considering the QTL effect of *FADS* gene variants. This study aimed to use lipidomics with a *FADS* gene-variant polymorphism as a covariate to examine the relationship between BD and PUFAs in the largest sample to date.

## Methods

### Subjects

The study recruited a total of 535 participants in the BD group and 107 in the control group from Fujita Health University and its affiliated hospitals. In the BD group, 179 of 535 individuals presented with type 1 BD (BD1), 313 with type 2 BD (BD2), and 43 with schizoaffective disorder (SA). In terms of clinical status, 117 individuals were in a depressive state, 28 in a manic state, and 390 were in remission. BD was diagnosed based on the Diagnostic and Statistical Manual of Mental Disorders 4^th^ edition (DSM-IV) by at least two experienced psychiatrists using medical records and conducting unstructured interviews with the participants and their families. Individuals with known intellectual disabilities were excluded.

Written informed consent was obtained from all participants after they received a complete description of the study. This study was approved by the ethics committees of Fujita Health University and each participating hospital.

### Fatty acid concentration measurement

Blood samples of the BD group were collected at the outpatient clinic, and plasma was extracted as soon as possible. In detail, vacuum tube blood collection was performed using Benoject II Vacuum Blood Collection Tubes VP-NA050K (containing EDTA-2Na), and 7 mL of blood was collected per tube. The blood samples were stored at 4 °C. To measure fatty acid concentrations, samples were centrifuged at 1000 g for 10 min at 4 °C to separate the plasma.

The separated plasma was stored at −80 °C, and the following 23 types of saturated, monounsaturated, and polyunsaturated fatty acids were analyzed using a triple quadrupole gas chromatograph mass spectrometer GCMS-TQ8040 (SHIMADZU CORPORATION Kyoto, Japan): ALA, EPA, DHA, LA, GLA, DGLA, AA, lauric acid, myristic acid, myristoleic acid, pentadecanoic acid, palmitic acid, palmitoleic acid, margaric acid, heptadecanoic acid, stearic acid, oleic acid, nonadecanic acid, arachidic acid, eicosenoic acid, eicosadienoic acid, behenic acid, and lignoceric acid.

To consider changes in the concentration of all fatty acids that fluctuate with diet and match the outcome of the previously reported QTL analysis [17–19], the fatty acid concentration ratio (each fatty acid concentration/total fatty acid concentration) was calculated from the measured fatty acid concentration. In this study, the n-3 and n-6 PUFAs (ALA, EPA, DHA, LA, GLA, DGLA, and AA) were measured and used as primary outcomes in the analysis. Further, other fatty acids were measured to calculate the concentration ratios.

### Genotyping

Typing was performed using Illumina HumanOmniExpressExome 1.2 chip for 520 subjects of the BD group and 72 subjects of the controls group. In addition, 15 subjects of the BD group and 35 subjects of the control group were further genotyped for rs174550 (*FADS* gene) using the TaqMan probe method. rs174550 was selected because rs174550 and top SNPs of a previous report [18] are in nearly absolute linkage disequilibrium (i.e., LA rs174547, *R*^2^ = 0.999; GLA rs174546, *R*^2^ = 0.999; DGLA rs174548, *R*^*2*^ = 1.0; AA rs174577, *R*^2^ = 0.9372; ALA rs509360, *R*^2^ = 0.9785; EPA rs174538, *R*^2^ = 1.0).

### Statistical analysis

First, a simple linear regression analysis was conducted using the diagnosis (BD group/control group) and rs174550 as independent variables. Next, multiple linear regression analysis was performed to compare the difference in PUFAs between the BD group and the control group. This analysis is the main analysis of this study. The ratio of each fatty acid (concentration of each PUFA/total fatty acid concentration) was used as the dependent variable. The independent variables included diagnosis (BD group /control group: primary outcome), sex, BMI, age, postprandial time (minutes), 4 °C storage time (minutes), hemolysis (yes/no), chyle (yes/no), dyslipidemia (yes/no), diabetes (yes/no), hypertension (yes/no), and rs174550 (CC/CT/TT). The level of significance was set at *p* < 0.05. The significance level for rs174550 was set at the level for the genome-wide significance (*p* < 5.0 × 10^−8^) because previous studies [15–19] referenced while selecting rs174550 were genome-wide association analyses; thus, we matched that level accordingly. Linear regression analyses were performed using R version 3.6.3.

## Results

Table 1 shows participants’ backgrounds. Significant differences between the BD group and control group were observed in, age, postprandial time, BMI, sex, and dyslipidemia status, all of which were covariates in the main analysis. Supplementary Table 1 shows the fatty acid concentration ratios and concentrations analyzed. Supplementary Table 2 depicts the results of the simple linear regression analysis of PUFA concentration ratio and diagnosis, PUFA concentration ratio, and rs174550. Table 2 shows the results of the diagnosis (BD group/control group)—the primary outcome in this study—and of rs174550 (CC/CT/TT) in the multiple linear regression analysis. Supplementary Table 3 shows all multiple linear regression analysis results.

**Table 1:** Characteristics of the BD and control groups.

	BD group		Control group		Statistical comparison		
	Mean ± SD	Range	Mean ± SD	Range	t/χ^2^	df	*p* value^*^
Age (years)	49.5 ± 14.7	18–90	42.9 ± 12.7	21–77	t = −4.32	640	**1.8** **×** **10**^**−5**^
Last meal (min)	300.5 ± 262.4	15–1490	456.3 ± 312.1	10–1080	t = 4.83	138	**4.0** **×** **10**^**−6**^
Preservation (min)	174.0 ± 92.6	20–465	178.7 ± 153.3	10–461	t = 0.30	122	0.76
Body mass index (kg/m^2^)	24.1 ± 4.8	13.1–47.3	22.6 ± 3.1	17.0–32.6	t = −3.90	234	**1.2** **×** **10**^**−4**^
Sex (N)	female: 308 male: 227		female: 43 male: 64		χ^2^ = 10.87	1	**9.8** **×** **10**^**−4**^
Hemolysis (N)	−: 459 +: 65 + +: 11		−: 92 +: 12 + +: 3		χ^2^ = 0.29	2	0.86
Chyle (N)	−: 415 +: 98 + +:22		−: 90 +: 17 + +:0		χ^2^ = 5.18	2	0.075
Dyslipidemia (N)	−: 509 +: 26		−: 95 +: 12		χ^2^ = 6.47	1	**0.011**
Diabetes (N)	−: 518 +: 17		−: 105 +: 2		χ^2^ = 0.53	1	0.47
Hypertension (N)	−: 496 +: 39		−: 99 +: 8		χ^2^ = 0.0046	1	0.95

BD bipolar disorder, SD standard deviation, N number, Last meal time since the last meal, Preservation: time of refrigeration after blood collection, t two independent samples t-tests, χ2 chi-square test, df degree of freedom.

*A *p* value of < 0.05 was considered statistically significant.

Significant *p* values are indicated in bold.

**Table 2:** Effects of diagnosis (BD/control) and the *FADS* gene variant (rs174550) on PUFA concentration ratios.

PUFA	Diagnosis (BD/control)^a^		rs174550 (C/T allele)^b^		Adjusted *R*^2^
	β	p value^*^	β	p value^**^	
LA	−0.36	**0.023**	0.045	0.59	0.16
GLA	0.025	0.10	−0.079	**< 2.0** **×** **10**^**−16**^	0.19
DGLA	0.0027	0.76	−0.0014	0.76	0.046
AA	−0.18	**0.013**	−0.36	**< 2.0** **×** **10**^**−16**^	0.25
ALA	0.00088	0.82	0.0043	0.035	0.050
EPA	−0.034	0.21	−0.038	0.0065	0.10
DHA	−0.19	0.052	−0.067	0.18	0.092

BD bipolar disorder, LA linoleic acid, GLA γ-linoleic acid, DGLA dihomo-γ-linolenic acid, AA arachidonic acid, ALA α-linoleic acid, EPA eicosapentaenoic acid, DHA docosahexaenoic acid.

^a^Each PUFA concentration ratio (%) in the BD group compared with that in the control group.

^b^Each PUFA concentration ratio (%) when possessing effect allele (C-allele).

*A *p* value of < 0.05 was considered statistically significant.

**A *p* value of < 5.0 × 10^−8^ was considered statistically significant.

Significant *p* values are indicated in bold.

The diagnostic status was significantly associated with the concentration ratios of LA (β = −0.36, *p* = 0.023) and AA (β = −0.18, *p* = 0.013) in multiple linear regression analysis. These results indicated that the BD group had significantly lower LA and AA concentration ratios. For other PUFAs, no association was found between BD diagnosis and concentration ratios.

The QTL SNP (rs174550), which was reported in previous studies [17–19], was significantly associated with the concentration ratios of GLA (β = −0.079, *p* < 2.0 × 10^−16^) and AA (β = −0. 36*, p* < 2.0 × 10^−16^). These mean that patients with the C-allele in rs174550, which was a risk allele of BD, had significantly lower concentration ratios of GLA and AA than those with the T-allele. However, compared with the findings of previous studies [17–19], other PUFAs were not associated with the genotypes in rs174550.

## Discussion

In this study, the BD group had significantly lower concentration ratios of LA and AA based on the analysis that took into account the QTL effect of SNP. This indicates the independent diagnostic effect of specific PUFAs on BD susceptibility. Furthermore, we conducted an ad hoc analysis to determine if the associations between PUFA and diagnosis differed depending on the BD subtype (BD1/BD2/SA) or status (depressive/manic/remission). Supplementary method 1 presents the detailed analysis methods. These results are shown in Supplementary Tables 4 and 5. Regarding the association between subtypes and LA, significant associations were found for BD1 and SA, with the same effect direction as observed in the overall BD group. Although BD2 was not significant, it showed the same effect direction. For AA, all subtypes showed significant associations with the same effect direction as observed in the overall BD group. Regarding the association between statuses and LA, significant associations were observed for all statuses, with the same effect direction as observed in the overall BD group. Regarding AA, significant associations were found in remission and manic statuses, with the same effect direction as observed in the overall BD group. Although the association with depressive status was not significant, it showed the same effect direction. Considerably, the effects of LA and AA observed in the overall BD group analysis are probably common across all BD subtypes or statuses.

In this study, we replicated the QTL effect on the concentrations of AA and GLA by the *FADS* SNP (rs174550), which was reported in various ancestries (Table 3) [17–19], although previous studies have shown that SNPs were associated with the concentrations of most PUFAs consisting of n-3 and n-6.

**Table 3:** Comparison of the QTL analysis results for rs174550 between this study and previous ones.

PUFA/study	The current study *N* = 645		Dorajoo et al. [18] *N* = 1361		Guan et al. [19] *N* = 8631	
	β	*p* value	β	*p* value	β	*p* value
LA	NS	NS	0.048	**1.5** **×** **10**^**−16**^	1.47	**4.4** **×** **10**^**−274**^
GLA	−0.079	**<2.0** **×** **10**^**−16**^	−0.52	**9.4** **×** **10**^**−171**^	−0.02	**6.0** **×** **10**^**−72**^
DGLA	NS	NS	−0.18	**1.2** **×** **10**^**−29**^	0.36	**7.4** **×** **10**^**−152**^
AA	−0.36	**<2.0** **×** **10**^**−16**^	−0.15	**2.1** **×** **10**^**−66**^	−1.69	**0**
ALA	NS	NS	0.38	${\mathbf{{1.1}}\,{\mathbf{\times}}\,{\mathbf{10}^{\mathbf{-}{\mathbf{16}}}}^{\mathbf{a}}}$	NA	NA
EPA	NS	NS	−0.045	${\mathbf{{2.2}}\,{\mathbf{\times}}\,{\mathbf{10}^{\mathbf{-}{\mathbf{16}}}}^{\mathbf{b}}}$	NA	NA
DHA	NS	NS	NS	NS	NA	NA

*N* number of samples, β: effect of C-allele on PUFA concentration ratio (%) in rs174550 (T > C effect allele: C-allele), *BD* bipolar disorder, *LA* linoleic acid, *GLA* γ-linoleic acid, *DGLA* dihomo-γ-linolenic acid, *AA* arachidonic acid, *ALA* α-linoleic acid, *EPA* eicosapentaenoic acid, *DHA* docosahexaenoic acid, NS = no significant difference, NA = not applicable.

^a^rs174547 data (rs174547 is in linkage disequilibrium with rs174550, *R*^2^ = 1.00).

^b^rs174538 data (rs174538 is in linkage disequilibrium with rs174550, *R*^2^ = 0.95).

Significant *p* values are indicated in bold.

**Fig. 1:**
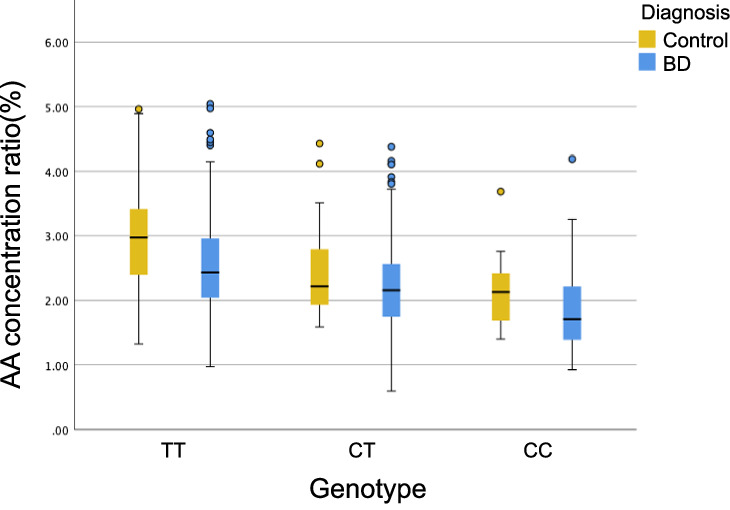
Comparison of fatty acid concentration ratio between the BD group and the control group by genotype for rs174550. AA arachidonic acid, BD bipolar disorder. The whiskers at the top/bottom represent the maximum/minimum value excluding outliers. The line that divides the box into two parts represents the median. The bottom of the box represents the first quartile. The top of the box represents the third quartile. The dots represent outliers. This figure shows that both the QTL effect of SNP (reduction in AA by genotype) and the diagnostic effect are independently involved in the BD risk by low AA concentration.

In the previous GWAS targeting BD [15], the C-allele of rs174550 was the risk allele for BD (OR = 1.17, *p* = 1.2 × 10^−8^). This finding supported the result that low GLA and AA concentrations in individuals harboring the C-allele were associated with BD, although other PUFAs, such as DHA and EPA, were not risk factors for BD. This also clarified the finding that BD risk by low AA concentration was explained both by the QTL effect of the SNP (decreased AA by genotype) and the diagnostic effect, which were independent of each other (AA, β = 0.070, *p* = 0.45; Fig. 1). By contrast, the low LA concentration was not influenced by the QTL effect but is mainly explained by the diagnostic effect. Such “non-significant” QTL effect is reasonable because LA concentration is mainly determined by diet (i.e., LA cannot be synthesized in the body), is in the upstream of the n-6 pathway, and is metabolized into GLA (significant QTL effect), DGLA, and AA (significant QTL effect) by *FADS*.

Compared with previous lipidomics studies of BD (Table 4), the sample size in the present study is one of the largest and thus provided the most powerful results. However, we could not obtain concordant and conclusive results similar to other studies because the sample size was not large enough to deny type I and II errors. In addition, previous studies did not consider the QTL effect of the *FADS* SNP, which is also a susceptible SNP to BD. Such a small sample size induces variable allele frequencies either in cases or controls, which may influence the QTL effect on PUFAs. Therefore, the QTL effect is one of the most important confounding factors when interpreting the lipidomics for BD.

**Table 4:** Comparison of the results of the current study and the lipidomic results of previous studies.

Study		Chiu et al., [7]	Sublette et al., [8]	Clayton et al., [9]	McNamara et al., [10]	Evans et al., [11]	Pomponi et al., [14]	Saunders et al., [12]	Koga et al., [13]	The current study
Sample		Erythrocyte	Plasma	Erythrocyte	Erythrocyte	Plasma	Plasma	Plasma	Plasma	Plasma
Unit		%	nmol/mL	mg/day	%	nmol/mL	nmol/mL	nmol/mL	μg/mL	%
Mood state (N)		Manic (20) Control (20)	Manic (10) Control (10)	BD (15) Control (15)	BD (20) MDD (20) Control (20)	BD (40) Control (18)	Depressed (21) Manic (9) Euthymic (12) Control (57)	BD (27) Control (31)	BD (83) Control (217)	BD (535) Control (107)
PUFA	LA	NS	NA	NA	NS	NS	↑	NA	↑	↓
	GLA	NS	NA	NA	NA	NS	NA	NA	↑	NS
	DGLA	NA	NA	NA	NS	↑	NA	NA	↑	NS
	AA	↓	NS	NA	NS	NS	↑	NS	↑	↓
	ALA	NA	NA	NA	NA	NS	↑	NS	↑	NS
	EPA	NS	NS	NS	NS	NS	↑	NS	↓	NS
	DHA	↓	NS	NS	↓	NS	↓	NS	↓	NS

*N* number, *NS* no significant difference, *NA* not applicable, ↑/↓: each PUFA concentration increases/decreases in the BD group compared to those in the control group.

The strength of this study is attributed to the largest sample size, and the “abnormality” of AA concentration is a good reason to explore the causal effect on BD susceptibility because the QTL effect was concordant with those in other QTL studies [17–19]. Because QTLs in other PUFAs, except GLA and AA, were not replicated in this study, we speculated the following reasons: (1) the sample size was small to detect the QTL effect and (2) previous QTL results showed that the direction of the effect on DGLA in rs174550 was contrasting between Caucasians and Han Chinese, which suggest population difference in the effect of rs174550 on DGLA. However, as mentioned above, we replicated the QTL effect of the SNP on AA and GLA, concordant with other large QTL analyses, and thus concluded concrete results at least for AA.

We believe that AA is important in elucidating the pathogenesis of BD because several associations between AA metabolism and BD were reported so far [20]. According to reports, antipsychotics, and mood stabilizers such as lithium salts, valproate, carbamazepine, and lamotrigine affected AA metabolism in the brain via N-methyl-D-aspartate receptors and D2 receptors, which the authors called the “arachidonic cascade hypothesis.” In this hypothesis, plasma AA is consumed in BD because of increased AA metabolism in the brain, and mood stabilizers decrease the turnover of AA in several brain phospholipids. Even in accordance with this hypothesis, as our study revealed compatible results that the plasma AA concentrations decreased, it supported the finding that impaired AA metabolism affects the pathogenesis of BD. Therefore, further studies investigating AA metabolism in the brain are essential and may elucidate the pathogenesis of BD.

To interpret this result, we stress important limitations. We could not control the effect of diet. Although we included the time from the last diet as a covariate in the model and applied the ratio (e.g., AA/all PUFAs) to control the diet, we could not follow the contents of the diet, and such is associated with PUFAs located in the upstream of this pathway (such as LA). Further studies should be performed to examine dietary intake and items that can be included in multiple linear regression analyses. Moreover, the effects of dyslipidemia and diabetes medications on PUFA concentrations have not been elucidated. However, these medications are important for the evaluation of PUFA levels. This is because dyslipidemia medications can affect cholesterol and triglyceride levels, and some diabetes treatments can affect lipid levels [21]. Supplementary Tables 6 and 7 present the breakdown of dyslipidemia and diabetes medications. We included information on dyslipidemia and diabetes in our analysis as they may influence blood lipid levels. However, treatment medications were not included in the analysis. This is because most patients with dyslipidemia were taking treatment medications. Similarly, most patients with diabetes were also on their medication. Treatment medication information was excluded from the analysis due to the strong correlation between dyslipidemia and its treatment medication (r = 0.97), as well as diabetes and its treatment medication (r = 0.95). To examine the impact of dyslipidemia and diabetes medications on PUFA concentrations, further research specifically targeting these medications must be performed. In addition, several participants in the BD group were taking mood stabilizers, antipsychotics, or both in this study. Therefore, the effects of these medications on PUFA were examined in this study. Supplementary Table 8 shows the types of mood stabilizers and antipsychotics used. To examine the effects of these medications on PUFA, a multiple linear regression analysis was conducted using groups taking antipsychotics (AP), lithium salts (Li), and anticonvulsants (AC) and a group not taking any of these medications. Supplementary method 2 and Supplementary Table 9 present the detailed analysis methods and results. The group not taking any of these medications was considered the reference group (no-medication group). The beta (β) coefficients of the concentration ratio of PUFA in groups taking AP (AP group), Li (Li group), AC (AC group), both AP and Li (AP + Li group), AP and AC (AP + AC group), Li and AC (Li + AC group) and AP, Li, and AC (AP + Li + AC group) were calculated. In particular, we focused on LA and AA, where significant associations were observed in the main analysis under the diagnostic categories of the independent variables. For LA, the AP and Li groups had positive β coefficients. The AC group and the two or more medications group had negative β coefficients, with a relatively large β coefficient and significant association observed particularly in the AP + Li + AC group. This finding indicates that the concomitant use of AP, Li, and AC decreased the LA concentration ratios. The effect of the diagnosis in reducing the LA concentration ratios observed in the main analysis may have been enhanced by the effect of reducing the LA concentration ratios present in the two or more medications group. However, understanding why a negative β coefficient was observed in the AP + Li group despite the positive β coefficients in the AP and Li groups, as well as why this trend is most pronounced in the AP + Li + AC group, is difficult. Furthermore, no studies have reported a relationship between these medications and LA so far. The effects of medications used in BD on LA should be clarified in future research with larger sample sizes. Regarding AA, there were no notably large β coefficients among the groups, nor were there any significant associations with AA. Therefore, the relationship between medications and AA was not clarified.

In conclusion, the BD group has significantly lower AA and LA concentration ratios than the control group. In addition, a strong QTL effect of the *FADS* SNP was found on AA and GLA; however, such an effect was independent of BD susceptibility. Therefore, AA and LA are good candidates for susceptibility to BD, and such QTL effect of the SNP should be included as a covariate to interpret the lipidomics for BD.

## Supplementary information

Supplementaryinformation

## Data Availability

All data relevant to the study are included in the article or uploaded as online supplementary information. The data generated in this study will be available from the corresponding author on reasonable request.
