# Supplementary material for: Association of plasma arachidonic acid levels with a bipolar disorder and the effects of a FADS gene variant
Source: Transl Psychiatry. 2024 Oct 14;14:435. doi: 10.1038/s41398-024-03141-1 (PMC11471766; doi:10.1038/s41398-024-03141-1)
Supplement: Supplementary file 1 — Supplementaryinformation [file 41398_2024_3141_MOESM1_ESM.docx]

**Supplementary information**

**Supplementary method 1**

**Effects of subtype (BD1/BD2/SA) or status (remission/depression/manic) on PUFA based on a multiple regression analysis**

This analysis included 642 participants (n = 535 in the BD group and n = 107 in the control group) who were the same participants in the main analysis. In the BD group, 179 of 535 individuals presented with type 1 BD (BD1), 313 with type 2 BD (BD2), and 43 with schizoaffective disorder (SA). In terms of clinical status, 117 individuals were in a depressive state, 28 in a manic state, and 390 were in remission. Linear regression analysis was performed using R, version 3.6.3. The ratio of each fatty acid (concentration of each PUFA/total fatty acid) was used as the dependent variable. The independent variables included subtype (control group/BD1/BD2/SA) or status (control group/remission/depressive/manic), sex, BMI, age, postprandial time (minutes), 4°C storage time (minutes), hemolysis (yes/no), chyle (yes/no), dyslipidemia (yes/no), diabetes (yes/no), hypertension (yes/no), and rs174550 (CC/CT/TT). A *p* value of < 0.05 was considered statistically significant.

**Supplementary method 2**

**Multiple regression analysis of the effects of antipsychotics and mood stabilizers on PUFA**

This analysis included 535 participants in the BD group, out of which, 502 were taking either AP, AC, Li, or a combination of these medications. In total, 33 BD participants were not taking AP, AC or Li. The medications included were those that had been taken continuously for >1 month before the blood collection.

The 535 BD participants were divided into eight groups: the AP, Li, AC, AP + Li, AP + AC, Li + AC, AP + Li + AC, and no medication groups. The AP group included participants who were taking AP alone.

Linear regression analysis was performed using R version 3.6.3. The ratio of each fatty acid (concentration of each PUFA/total fatty acid) was used as the dependent variable. The independent variables included BD medications (AP group/Li group/AC group/AP + Li group/AP + AC group/Li + AC group/AP + Li + AC group/no medication group), sex, BMI, age, postprandial time (minutes), 4°C storage time (minutes), hemolysis (yes/no), chyle (yes/no), dyslipidemia (yes/no), diabetes (yes/no), hypertension (yes/no), and rs174550 (CC/CT/TT). Values of *p* < 0.05 were accepted as indicating statistical significance.**Supplementary Figure**

**n-3 PUFAs**

**n-6 PUFAs**

elongation

elongation

**C18:3n-3(ALA)**

**C18:2n–6(LA)**

**FADS2**

**C20:2n-6**

**C20:3n-3**

**C18:4n-3**

**C18:3n-6(GLA)**

elongation

**FADS2**

**FADS2**

**C20:4n-3**

**C20:3n-6(DGLA)**

**FADS1**

**C20:5n-3(EPA)**

**C20:4n-6(AA)**

elongation

**C22:4n-6**

**C22:5n-3**

elongation

**C24:5n-3**

**C24:4n-6**

**FADS2**

**C24:6n-3**

**C24:5n-6**

β-oxdation

**C22:6n-3(DHA)**

**C22:5n-6**

Supplementary Figure 1.

Metabolic pathway diagram for n-3 and n-6 PUFAs.

PUFAs: polyunsaturated fatty acids, FADS: fatty acid desaturase, LA: linoleic acid, GLA: γ-linoleic acid, DGLA: dihomo-γ-linolenic acid, AA: arachidonic acid, ALA: α-linoleic acid, EPA: eicosapentaenoic acid, DHA: docosahexaenoic acid

The blue arrow represents the pathway involving FADS1.

The red arrow represents the pathway involving FADS2.

The black arrow represents the pathway involving other enzymes.

**Supplementary Table**

|  |  | BD group (n = 535) | | Control group (n = 107) | |
| --- | --- | --- | --- | --- | --- |
|  |  | Median  Concentration ratio [%]  (Measured concentration [μg/mL]) | 1^st^–3^rd^ quartiles | Median  Concentration ratio [%]  (Measured concentration [μg/mL]) | 1^st^–3^rd^ quartiles |
| n-3 PUFAs | ALA | 0.040  (3.59) | 0.021–0.060  (2.37–6.28) | 0.037  (3.38) | 0.028–0.055  (2.12–5.10) |
|  | EPA | 0.25  (27.6) | 0.17–0.43  (17.9–42.1) | 0.28  (25.7) | 0.18–0.44  (17.1–36.7) |
|  | DHA | 2.05  (213.7) | 1.55–2.80  (161.8–274.9) | 2.16  (179.4) | 1.68–2.81  (148.0–237.8) |
| n-6 PUFAs | LA | 4.58  (459.0) | 3.63–5.58  (396.8–539.1) | 4.81  (434.7) | 4.01–5.82  (370.3–485.8) |
|  | GLA | 0.20  (20.5) | 0.13–0.31  (12.2–32.6) | 0.19  (15.3) | 0.10–0.27  (8.68–23.0) |
|  | Eicosadienoic acid | 0.010  (1.12) | 0.007–0.018  (0.70–1.73) | 0.018  (1.45) | 0.012–0.021  (0.93–2.02) |
|  | DGLA | 0.22  (21.7) | 0.16–0.27  (16.3–29.4) | 0.22  (18.6) | 0.17–0.26  (13.9–23.4) |
|  | AA | 2.18  (221.5) | 1.74–2.64  (179.0–267.5) | 2.39  (209.6) | 2.00–2.94  (181.6–239.6) |
| n-5 PUFAs | Myristoleic acid | 0.019  (1.83) | 0.010–0.031  (0.88–3.58) | 0.014  (1.11) | 0.008–0.023  (0.88–3.58) |
| n-7 PUFAs | Palmitoleic acid | 0.92  (90.2) | 0.68–1.21  (61.7–134.5) | 0.78  (62.1) | 0.61–1.03  (48.7–90.3) |
|  | Heptadecanoic acid | 0.023  (2.03) | 0.012–0.032  (1.22–3.29) | 0.021  (1.81) | 0.014–0.030  (1.07–2.91) |
| n-9 PUFAs | Oleic acid | 6.07  (602.8) | 4.92–7.29  (477.2–770.0) | 5.59  (496.9) | 4.88–6.42  (393.6–583.6) |
|  | Eicosenoic acid | 0.092  (8.44) | 0.060–0.12  (6.14–11.8) | 0.090  (7.39) | 0.067–0.10  (5.19–9.54) |
| SAFAs | Lauric acid | 0.042  (4.24) | 0.022–0.079  (2.07–8.56) | 0.038  (3.46) | 0.019–0.071  (1.70–6.48) |
|  | Myristic acid | 2.80  (279.6) | 2.04–3.58  (177.5–410.0) | 2.34  (189.3) | 1.73–2.86  (139.6–285.5) |
|  | Pentadecanoic acid | 0.41  (41.5) | 0.33–0.50  (31.8–55.1) | 0.41  (33.4) | 0.33–0.47  (26.8–40.9) |
|  | Palmitic acid | 61.2  (6360) | 59.4–63.0  (4784–8086) | 59.6  (5186) | 57.5–60.9  (4234–6147) |
|  | Margaric acid | 0.64  (65.4) | 0.54–0.75  (52.1–81.5) | 0.68  (56.9) | 0.56–0.77  (48.6–66.2) |
|  | Stearic acid | 17.4  (1872) | 13.8–20.8  (1138–2754) | 19.8  (1716) | 17.2–22.0  (1394–2266) |
|  | Nonadecanoic acid | 0.034  (3.51) | 0.025–0.047  (2.75–4.26) | 0.037  (3.11) | 0,030–0.046  (2.57–3.76) |
|  | Arachidic acid | 0.13  (14.2) | 0.11–0.17  (10.5–20.1) | 0.14  (12.3) | 0.11–0.18  (9.54–17.0) |
|  | Behenic acid | 0.032  (3.07) | 0.020–0.049  (2.02–4.84) | 0.030  (2.47) | 0.022–0.045  (1.76–4.13) |
|  | Lignoceric acid | 0.010  (1.08) | 0.007–0.018  (0.70–1.79) | 0.009  (0.75) | 0.006–0.013  (0.45–1.34) |
| Total | | 100  (10561) | 25-75  (7856–3239) | 100  (8758) | 25-75  (7219–10379) |

Supplementary Table 1. Concentration of each fatty acid.

PUFAs: polyunsaturated fatty acids, SAFAs: saturated fatty acids, BD: bipolar disorder, LA: linoleic acid, GLA: γ-linoleic acid, DGLA: dihomo-γ-linolenic acid, AA: arachidonic acid, ALA: α-linoleic acid, EPA: eicosapentaenoic acid, DHA: docosahexaenoic acid

| PUFA | Diagnosis (BD/control) ^a^ | | | rs174550 (C/T allele) ^b^ | | |
| --- | --- | --- | --- | --- | --- | --- |
|  | β | *p* value^*^ | R^2^ | β | *p* value ^**^ | R^2^ |
| LA | −0.44 | **0.0076** | 0.011 | 0.098 | 0.26 | 0.0020 |
| GLA | 0.034 | **0.031** | 0.0072 | −0.079 | **<2.0 × 10^−16^** | 0.14 |
| DGLA | 0.011 | 0.19 | 0.0027 | −0.0020 | 0.66 | 0.00030 |
| AA | −0.28 | **0.00025** | 0.021 | −0.36 | **<2.0 × 10^−16^** | 0.12 |
| ALA | 0.0042 | 0.28 | 0.0018 | 0.0057 | 0.0052 | 0.012 |
| EPA | −0.0064 | 0.81 | 9.6 × 10^−5^ | −0.037 | 0.0057 | 0.012 |
| DHA | −0.075 | 0.44 | 0.00092 | −0.056 | 0.27 | 0.0019 |

Supplementary Table 2. Univariate analysis of polyunsaturated fatty acids and diagnosis or the FADS genes.

BD: bipolar disorder, LA: linoleic acid, GLA: γ-linoleic acid, DGLA: dihomo-γ-linolenic acid, AA: arachidonic acid, ALA: α-linoleic acid, EPA: eicosapentaenoic acid, DHA: docosahexaenoic acid

^a^ Each PUFA concentration ratio (%) in the BD and control groups

^b^ Each PUFA concentration ratio (%) when possessing effect allele (C-allele)

* A *p* value of < 0.05 was considered statistically significant.

** A *p* value of < 5.0 × 10^−8^ was considered statistically significant.

Significant *p* values are indicated in bold.

|  | Sex | | BMI | | Age | | Postprandial time  (minutes) | | 4°C storage time (minutes) | | Hemolysis | | Chyle | | Dyslipidemia | | Diabetes | | Hypertension | | Diagnosis (BD/control) | | rs174550 (C/T allele) | | |
| --- | --- | --- | --- | --- | --- | --- | --- | --- | --- | --- | --- | --- | --- | --- | --- | --- | --- | --- | --- | --- | --- | --- | --- | --- | --- |
|  | β | *P* value | β | *P* value | β | *P* value | β | *P* value | β | *P* value | β | *P* value | β | *P* value | β | *P* value | β | *P* value | β | *P* value | β | *P* value | β | *P* value |  |
| LA | **−**0.21 | 0.090 | −0.067 | 8.3×  10^−7^ | −0.016 | 0.00020 | 0.19 | 0.55 | −3.16 | 0.00017 | 0.092 | 0.52 | −0.082 | 0.52 | −0.13 | 0.60 | −0.14 | 0.68 | −0.70 | 0.0026 | −0.36 | 0.023 | 0.045 | 0.59 |  |
| GLA | −0.0041 | 0.73 | 0.0059 | 8.8× 10^−6^ | 0.00054 | 0.20 | 0.060 | 0.049 | −0.045 | 0.58 | −0.0097 | 0.48 | 0.011 | 0.39 | 0.054 | 0.024 | −0.043 | 0.20 | −0.053 | 0.019 | 0.025 | 0.10 | −0.079 | < 2.0 × 10^−16^ |  |
| DGLA | −0.0055 | 0.43 | 0.0035 | 3.5× 10^−6^ | −0.00011 | 0.64 | −0.0035 | 0.84 | −0.069 | 0.14 | 0.0050 | 0.53 | −0.0093 | 0.19 | 0.0089 | 0.52 | −0.019 | 0.31 | −0.049 | 0.00018 | 0.0027 | 0.76 | −0.0014 | 0.76 |  |
| AA | −0.13 | 0.016 | −0.030 | 7.4× 10^−7^ | −0.0082 | 2.8× 10^−5^ | 0.10 | 0.47 | −1.02 | 0.0065 | −0.0034 | 0.96 | −0.19 | 0.00073 | 0.39 | 0.00049 | −0.097 | 0.53 | −0.099 | 0.34 | −0.18 | 0.013 | −0.36 | < 2.0 × 10^−16^ |  |
| ALA | 0.0080 | 0.78 | −2.7× 10^−5^ | 0.93 | 9.8× 10^−5^ | 0.36 | 0.0088 | 0.25 | −0.045 | 0.031 | 2.80×  10^−5^ | 0.99 | 0.015 | 2.1×  10^−6^ | −0.0039 | 0.52 | −0.0094 | 0.27 | −0.0084 | 0.14 | 0.00088 | 0.82 | 0.0043 | 0.035 |  |
| EPA | −0.0042 | 0.84 | 0.00048 | 0.83 | 0.0051 | 5.2× 10^−12^ | 0.045 | 0.39 | −0.31 | 0.027 | 0.00057 | 0.98 | -0.038 | 0.078 | 0.087 | 0.037 | -0.089 | 0.12 | −0.021 | 0.60 | −0.034 | 0.21 | −0.038 | 0.0065 |  |
| DHA | −0.14 | 0.059 | −0.017 | 0.036 | 0.015 | 2.0× 10^−8^ | 0.13 | 0.48 | −1.47 | 0.0042 | −0.037 | 0.67 | −0.19 | 0.016 | 0.11 | 0.45 | −0.34 | 0.11 | −0.15 | 0.29 | −0.19 | 0.052 | −0.067 | 0.18 |  |

Supplementary Table 3. Effects of independent variables on PUFA concentration ratios

β: Each PUFA concentration ratio (%) in the subtype groups compared with that in the control group, LA: linoleic acid, GLA: γ-linoleic acid, DGLA: dihomo-γ-linolenic acid, AA: arachidonic acid, ALA: α-linoleic acid, EPA: eicosapentaenoic acid, DHA: docosahexaenoic acid

|  | BD1 (n = 179) | | BD2 (n = 313) | | SA (n = 43) | |
| --- | --- | --- | --- | --- | --- | --- |
|  | β | *P* value | β | *P* value | β | *P* value |
| LA | −0.53 | **0.0053** | −0.26 | 0.11 | −0.70 | **0.014** |
| GLA | 0.017 | 0.34 | 0.030 | 0.06 | 0.0097 | 0.73 |
| DGLA | −0.0072 | 0.49 | 0.0094 | 0.31 | −0.026 | 0.10 |
| AA | −0.20 | **0.017** | −0.16 | **0.035** | −0.31 | **0.013** |
| ALA | −0.0046 | 0.32 | 0.0036 | 0.37 | −0.0034 | 0.62 |
| EPA | −0.027 | 0.39 | −0.040 | 0.15 | 0.0090 | 0.85 |
| DHA | −0.17 | 0.14 | −0.18 | 0.068 | −0.34 | 0.053 |

Supplementary Table 4. Effects of subtypes (BD1/BD2/SA) on PUFA concentration ratios on the multiple regression

n: number of samples, β: Each PUFA concentration ratio (%) in the subtype groups compared with that in the control group, LA: linoleic acid, GLA: γ-linoleic acid, DGLA: dihomo-γ-linolenic acid, AA: arachidonic acid, ALA: α-linoleic acid, EPA: eicosapentaenoic acid, DHA: docosahexaenoic acid

Significant *p* values are indicated in bold.

|  | depressive (n = 117) | | manic (n = 28) | | remission (n = 390) | |
| --- | --- | --- | --- | --- | --- | --- |
|  | β | *P* value | β | *P* value | β | *P* value |
| LA | −0.35 | **0.075** | −0.83 | **0.012** | −0.33 | **0.043** |
| GLA | 0.023 | 0.22 | 0.013 | 0.69 | 0.027 | 0.10 |
| DGLA | 0.00015 | 0.99 | 0.0017 | 0.93 | 0.0036 | 0.69 |
| AA | −0.087 | 0.32 | −0.39 | **0.0092** | −0.20 | **0.0073** |
| ALA | −0.00045 | 0.93 | −0.0010 | 0.22 | 0.0021 | 0.61 |
| EPA | −0.024 | 0.47 | 0.011 | 0.84 | −0.040 | 0.15 |
| DHA | −0.090 | 0.45 | −0.28 | 0.17 | −0.22 | **0.030** |

Supplementary Table 5. Effects of bipolar disorder statuses (depressive/manic/remission) on PUFA concentration ratios on the multiple regression

n: number of samples, β: Each PUFA concentration ratio (%) in the status compared with that in the control group, LA: linoleic acid, GLA: γ-linoleic acid, DGLA: dihomo-γ-linolenic acid, AA: arachidonic acid, ALA: α-linoleic acid, EPA: eicosapentaenoic acid, DHA: docosahexaenoic acid

Significant *p* values are indicated in bold.

|  | BD group (n = 535) | | Control group (n = 107) | |
| --- | --- | --- | --- | --- |
| Medications for dyslipidemia | Number of participants ^a^  (%) ^b^ | Mean daily dose  　　　 (mg) | Number of participants ^a^  (%) ^c^ | Mean daily dose  (mg) |
| Rosuvastatin | 3  (0.56) | 2.5 | 3  (2.8) | 4.2 |
| Pravastatin | 3  (0.56) | 10 | 1  (0.93) | 10 |
| Atorvastatin | 3  (0.56) | 10 | 1  (0.93) | 10 |
| Pitavastatin | 1  (0.19) | 2 | 1  (0.93) | 1 |
| Fenofibrate | 3  (0.56) | 177.8 | 1  (0.93) | 160 |
| Bezafibrate | 1  (0.19) | 200 | 1  (0.93) | 400 |
| Ezetimibe | 0  (0) |  | 1  (0.93) | 10 |
| Icosapentate | 1  (0.19) | 1800 | 0  (0) |  |
| Omega-3 fatty acid | 1  (0.19) | 2 | 0  (0) |  |
| No medication | 0  (0) |  | 3  (2.8) |  |
| No information on medications ^d^ | 11  (2.1) |  | 1  (0.93) |  |

Supplementary Table 6. Types of dyslipidemia medications

^a^ Number of participants taking each medication

^b^ Percentage of participants taking each type of medication in the BD group

^c^ Percentage of participants taking each type of medication in the control group

^d^ Participants with unknown medication status

BD: bipolar disorder

|  | BD　group (n = 535) | | Control group (n = 107) | |
| --- | --- | --- | --- | --- |
| Medications for diabetes | Number of participants ^a^  (%) ^b^ | Mean daily dose  (mg) | Number of participants ^a^  (%) ^c^ | Mean daily dose  (mg) |
| Metformin | 4  (0.75) | 750 | 0  (0) |  |
| Voglibose | 4  (0.75) | 0.83 | 0  (0) |  |
| Pioglitazone | 2  (0.37) | 15 | 1  (0.93) | 30 |
| Alogliptin | 2  (0.37) | 13.8 | 1  (0.93) | 25 |
| Sitagliptin | 1  (0.19) | 50 | 0  (0) |  |
| Mitiglinide | 1  (0.19) | 10 | 0  (0) |  |
| Glimepiride | 2  (0.37) | 1 | 0  (0) |  |
| Empagliflozin | 2  (0.37) | 10 | 0  (0) |  |
| Insulin glargine | 1  (0.19) | 14 (units) | 0  (0) |  |
| Insulin aspart | 1  (0.19) | 18 (units) | 0  (0) |  |
| No medication | 0  (0) |  | 1  (0.93) |  |
| No information on medications ^d^ | 7  (1.3) |  | 1  (0.93) |  |

Supplementary Table 7. Types of diabetes medications

^a^ Number of participants taking each medication

^b^ Percentage of participants taking each type of medication in the BD group

^c^ Percentage of participants taking each type of medication in the control group

^d^ Participants with unknown medication status

BD: bipolar disorder

| **Antipsychotics** | Number of participants taking medications  (%) | Mean daily dose  (mg) |
| --- | --- | --- |
| Aripiprazole | 101  （18.9） | 9.17 |
| Quetiapine | 95  (17.8) | 214.3 |
| Olanzapine | 63  (11.8) | 7.37 |
| Risperidone | 38  (7.10) | 2.68 |
| Levomepromazine | 20  (3.74) | 39.5 |
| Blonanserin | 14  (2.62) | 5.93 |
| Chlorpromazine | 9  (1.68) | 51.5 |
| Perospirone | 7  (1.31) | 7.71 |
| Haloperidol | 6  (1.12) | 3.33 |
| Aripiprazole-LAI | 4  (0.75) | 375 |
| Quetiapine sustained-release formulation | 1  (0.19) | 300 |
| Paliperidone | 1  (0.19) | 12 |
| Asenapine | 1  (0.19) | 20 |
| Zotepine | 1  (0.19) | 30 |
|  |  |  |
| **Mood stabilizer** |  |  |
| Lithium | 257  (48.0) | 630.7 |
| Valproic acid | 196  (36.6) | 631.1 |
| Lamotrigine | 93  (17.4) | 186.8 |
| Carbamazepine | 14  (2.62) | 431.4 |

Supplementary Table 8. Antipsychotics and mood stabilizers in the bipolar disorder group

|  | No medication  n = 33 | AP group  n = 59 | | Li group  n = 22 | | AC group  n = 19 | | AP + Li group  n = 21 | | AP + AC group  n = 27 | | Li + AC group  n = 159 | | AP + Li + AC group  n = 195 | | |
| --- | --- | --- | --- | --- | --- | --- | --- | --- | --- | --- | --- | --- | --- | --- | --- | --- |
|  | − | β | *P* value | β | *p* value | β | *p* value | β | *p* value | β | *p* value | Β | *p* value | β | *p* value |  |
| LA | − | 0.052 | 0.87 | 0.035 | 0.93 | −0.38 | 0.37 | −0.70 | 0.080 | −0.43 | 0.25 | −0.53 | 0.063 | −0.97 | 0.00061 |  |
| GLA | − | 0.053 | 0.12 | 0.0078 | 0.85 | 0.037 | 0.40 | 0.029 | 0.49 | 0.062 | 0.11 | 0.020 | 0.49 | 0.043 | 0.14 |  |
| DGLA | − | 0.017 | 0.38 | 0.0087 | 0.71 | 0.0073 | 0.77 | 0.0020 | 0.93 | 0.020 | 0.35 | −0.0076 | 0.64 | −0.0097 | 0.55 |  |
| AA | − | 0.12 | 0.37 | −0.093 | 0.61 | −0.019 | 0.92 | −0.05 | 0.77 | 0.13 | 0.43 | 0.10 | 0.45 | −0.060 | 0.63 |  |
| ALA | − | 0.015 | 0.076 | −0.0040 | 0.70 | 0.0026 | 0.81 | −0.0017 | 0.87 | 0.00080 | 0.93 | −0.0076 | 0.30 | −0.014 | 0.048 |  |
| EPA | − | −0.053 | 0.36 | −0.034 | 0.64 | −0.097 | 0.20 | −0.088 | 0.21 | −0.084 | 0.21 | −0.070 | 0.16 | −0.068 | 0.17 |  |
| DHA | − | −0.16 | 0.43 | −0.13 | 0.60 | −0.31 | 0.24 | −0.21 | 0.41 | −0.20 | 0.40 | −0.42 | 0.020 | −0.45 | 0.0098 |  |

Supplementary Table 9. Effects of the medications on PUFA based on the multiple regression analysis. n: number of samples, β: PUFA concentration ratio in the group not taking oral medications, AP: antipsychotics, Li: lithium, AC: anticonvulsant, LA: linoleic acid, GLA: γ-linoleic acid, DGLA: dihomo-γ-linolenic acid, AA: arachidonic acid, ALA: α-linoleic acid, EPA: eicosapentaenoic acid, DHA: docosahexaenoic acid
